# Halochromic Bacterial Cellulose/Anthocyanins Hybrid Polymer Film with Wound-Healing Potential

**DOI:** 10.3390/polym16162327

**Published:** 2024-08-16

**Authors:** Ganna Zubova, Hanna Melnyk, Iryna Zaets, Tetyana Sergeyeva, Olesia Havryliuk, Sergiy Rogalsky, Lyudmila Khirunenko, Leonid Zaika, Tetiana Ruban, Svitlana Antonenko, Natalia Kozyrovska

**Affiliations:** 1 Institute of Molecular Biology and Genetics, National Academy of Sciences of Ukraine, 150, Zabolotnogo Str., 03143 Kyiv, Ukraine; h.b.melnyk@imbg.org.ua (H.M.); i.e.zaets@imbg.org.ua (I.Z.); t.a.sergeyeva@imbg.org.ua (T.S.); l.a.zaika@imbg.org.ua (L.Z.); s.v.antonenko@imbg.org.ua (S.A.); n.o.kozyrovska@imbg.org.ua (N.K.); 2 Department of Extremophilic Microorganisms Biology, D. K. Zabolotny Institute of Microbiology and Virology of the National Academy of Sciences of Ukraine, 154 Zabolotnogo St., 03143 Kyiv, Ukraine; o.havryliuk@imv.org.ua; 3 V.P. Kukhar Institute of Bioorganic Chemistry and Petrochemistry, National Academy of Sciences of Ukraine, Academician Kukhar Str. 1, 02094 Kyiv, Ukraine; rogalsky@bpci.kiev.ua; 4 Institute of Physics, National Academy of Sciences of Ukraine, 46 Nauky Ave., 03680 Kyiv, Ukraine; lukh@iop.kiev.ua

**Keywords:** anthocyanins, natural pH-sensor, microbial infection, bacterial cellulose, hybrid polymer, wound healing

## Abstract

Polymer-based dressings deriving from natural biomaterials have advantages such as nontoxicity, biocompatibility, and mechanical stability, which are essential for efficient wound healing and microbial infection diagnostics. Here, we designed a prototype of an intelligent hydrogel dressing on the base of bacterial cellulose (BC) for monitoring wound microbial infection due to the uploaded natural pH dye-sensor, anthocyanins (ANC) of elderberry fruit (*Sambucus nigra* L.). The highest sensor responses to bacterial metabolites for ANC immobilized to BC were observed at pH 5.0 and 6.0. The detection limit of the sensor signals was 3.45 A.U., as it was evaluated with a smartphone-installed application. The FTIR spectral analysis of the hybrid BC/ANC hydrogel films has proved the presence of anthocyanins within the BC matrix. Hybrid films differed from the control ones by thicker microfibrils and larger pores, as detected with scanning electron microscopy. Halochromic BC/ANC films exhibited antimicrobial activities mainly against gram-positive bacteria and yeast. They showed no cytotoxicity for the in vitro human cell lines and mouse fibroblasts within a selected range of anthocyanin concentrations released from the BC/ANC film/dressing prototype. Compared to the control, the in vitro healing test showed overgrowth of primary mouse fibroblasts after applying 0.024–2.4 µg/mL ANC.

## 1. Introduction

Bacterial infection is the most common and unavoidable problem in wound healing, as it can cause inflammatory reactions in a wound and delay healing [1]. To date, direct enumeration of microorganisms in a wound, i.e., cultivation of microorganisms and counting colonies, remains the most traditional approach in clinical practice. However, the method requires a long experimental time to cultivate microorganisms [2]. In addition to colony counting, immunoassay and polymerase chain reaction are also used to detect and identify contaminating or pathogenic microorganisms [3,4,5]. The detection methods also include flow cytometry [6,7], spectroscopy-based methods of Fourier Transform InfraRed spectroscopy (FTIR), Raman spectroscopy, and hyperspectral imaging [8,9,10]. Despite being accurate, reliable, and sensitive to the single-cell level [11], these methods are expensive and time-consuming, requiring centralized laboratories, trained personnel, sample pretreatment, and multistep processing. To overcome these limitations, microflora sensors based on paper platforms [12,13], nanoplasmonic [14], electrical [15], and optical devices [16], as well as potentiometric [17], fluorometric [18] and colorimetric [19] pH sensors were developed. The most recent of them are described in the review by Youseff et al. [20].

For colorimetric detection of pH change caused by microbial infection, sensitive dyes are embedded into the auxiliary material to detect pH changes visually or by using image processing without embedding electronics. A fundamental limitation of dyes is their inability to bind to commercial wound dressings covalently, making the cellulosic polymer with open functional groups a promising matrix for delivering pH-sensitive indicators. Hydrated bacterial cellulose (BC), a matrix for antimicrobial and healing agents [21,22,23,24,25,26,27], has recently gained popularity as a means of treating wound surfaces and thermal skin burns of I-II degrees, as its high conformability provides close contact between the wound tissue and the dressing, relieving pain, stopping bleeding, and potentially enabling sensitive biomarker detection when integrating sensing elements into BC films. BC is produced by various bacteria (*Komagataeibacter* spp. are commercially used), resulting in a hydrated film with a unique three-dimensional hierarchical structure that resembles skin [28]. This allows various systems to be introduced while maintaining high mechanical tensile strength and elasticity of dressings [21,22,24,25]. A seminal recent review [29] demonstrates BC use for the design of wound dressings, including various production strains and their biosynthetic mechanisms.

BC can provide the desired properties for wearable sensors and smart electronics as a natural polymer. The potential of bacterial cellulose-based electrochemical biosensors is well-reviewed in [30]. Among others, electrochemical biosensors for sensitive *Staphylococcus aureus* detection were successfully developed, using BC as an effective matrix for high-density phage particle immobilization [31]. Incorporating pH-sensitive sensors into cellulose hydrogels may combine the healing properties of hydrogel wound dressings with diagnostic ones. To develop a colorimetric method for monitoring wound infection, we selected natural dye—anthocyanins (from the extract of elderberries, *Sambucus nigra* L.), which change color in response to pH changes in the microenvironment. Anthocyanins are nontoxic flavonoids abundant in plants [32]. By structure, they are substituted glycosides and acyl glycosides of 2-phenylbenzopyrylium salts, i.e., anthocyanidins. Structurally, anthocyanins consist of a C6-C3-C6 framework called the flavylium cation [33], and, depending on the number and position of the attached hydroxyl and/or methyl ester groups, they are of different types. Elderberry anthocyanins mainly consist of cyanidin-sambubiosides and cyanidin-glucosides (90.1% of identified anthocyanins) [34]. Anthocyanin content is the highest in elderberry, which has potent antioxidant and radical scavenging activity, and due to these properties, they have potential in wound healing [35,36,37] and other applications [38]. Remarkably, elderberries possess the immune-stimulating potential and improved functions under a SARS-CoV-2 pandemic, i.e., these fruits are not toxic in therapeutic doses but need control because of probable cytokine overproduction [39].

Recently, BC, containing anthocyanins, was used to design halochromic eco-friendly intelligent film packages to preserve fresh products [40,41]. Anthocyanin extract, as a green metallochromic sensor uploaded into BC, was also used to detect aluminum [Al(III)) or cuprum (Cu(II)] ions in solution and solid state [42,43]. Considering increasing environmental awareness and public concern about pollution, using BC as a natural matrix that is easy to functionalize for a pH-sensitive dye with a set of biological activities, promoting rapid skin regeneration, can fill a gap in wound healing theranostics.

This study aims to develop an eco-friendly antimicrobial wound dressing that uses rapid, efficient, affordable, and noninvasive visual and analytical methods to predict microbial infection in a primary wound bed and evaluate the healing potential of halochromic prototype dressing in the in vitro experiments. The combination of pH sensitivity and healing potential of elderberry extract with BC might provide new material for wound healing, online monitoring of on-site wound infection, and the design of multiplexed theranostic smart dressings.

## 2. Materials and Methods

### 2.1. Microorganisms and Culturing

The five bacterial strains and a kombucha microbial community were used for the research. *Bacillus subtilis* B-901 and *Candida albicans* UCM-1918, were obtained from the Ukrainian Collection of Microorganisms. *Pseudomonas aeruginosa* ATCC 10145, *Staphylococcus aureus* ATCC 23235, and *Escherichia coli* ATCC 25922 were obtained from the American Type Culture Collection (Manassas, VA, USA). Kombucha Multimicrobial Community (KMC) (bacteria and yeast) of the IMBG-1 ecotype was used from the collection of the Institute of Molecular Biology and Genetics of the National Academy of Sciences of Ukraine. The microorganisms were cultured in LB broth at 28 °C overnight. The tea medium for the KMC growing consisted of the following components: green leaf tea (0.5%), white sugar (7.0%), and tap boiled water to the required volume. The cultivation temperature for KMC was 28 °C within one week.

### 2.2. Selection of Plant Material and Preparation of Anthocyanin Extract (AE)

This study used the widespread black elder (*Sambucus nigra* L.) from the spontaneous flora of Ukraine, near Kyiv. This plant contains anthocyanin pigments [32]. Elderberry fruits were collected in September 2023 and frozen at −20 °C. The AE of 20% of the berries was prepared in ethyl alcohol (40°) with exposure for 3 h. The alcohol extract was squeezed out of the crushed berries, passed through a sieve, filtered through paper, and stored in a black glass bottle. For various manipulations, AE was diluted with phosphate-citrate buffer solutions.

### 2.3. Anthocyanin Characterization

The anthocyanin extract was diluted (dilution factor = 10) using pH 4 to 12 phosphate-citrate buffer solutions. The absorption spectra of diluted samples were measured in triplicate using a spectrophotometer (UV-VIS) NanoDrop ND-1000 (NanoDrop Technologies Inc, Wilmington, DE, USA) from 280 to 800 nm.

The total monomeric anthocyanin content in AE was measured using a spectrophotometric pH differential method according to the AOAC official method 2005.02 [44]. To measure anthocyanin content in the sample, two 50 μL-aliquots were diluted with 150 μL 0.025 M potassium chloride, pH 1.0, and 0.4 M sodium acetate, pH 4.5, buffers, accordingly, and dilutions were left to equilibrate for 15 min. The dilution factor, corresponding to the spectrophotometer’s linear range (A < 1.5), was 4. The absorption peak was detected at the wavelength 520 nm (n = 6). The haze appearance, which could affect the results, was corrected by reading at a 700 nm wavelength.

To calculate the absorbance of the diluted sample (*A*), the following Equation (1) was used:

(1) $$A=(A_{520}-A_{700}{)}_{pH\;1.0}-(A_{520}-A_{700}{)}_{pH\;4.5}$$

Monomeric anthocyanin pigment concentration (*MAPC*) (for elderberry extracts, cyanidin-3-*O*-glycoside (C-3-*O*-glu) is mentioned to be dominant [34]) via the next Equation (2):

(2) $$MAPC\;\left(\frac{mg}{L}\right)=\frac{A\times MW\times DF\times 1000}{\varepsilon \times l}\;$$

where, *A*—absorption of the sample, *MW*—C-3-*O*-glu molar weight, *DF*—dilution factor, ε—C-3-*O*-glu molar absorptivity, l = 0.1 cm—spectrophotometer path length. To validate the *MAPC* in the raw elderberry juice, C-3-*O*-glu concentration in the ethanol elderberry extract was multiplied by 3.5 (dilution factor) and the absolute *MAPC* error was calculated. Statistical error calculations were performed by Equations (3)–(5) listed below:

(3) $$\Delta A_{abs}=\Delta A_{520\left(pH1.0\right)}+\Delta A_{700\left(pH\;1.0\right)}+\Delta A_{520\;(pH\;4.5)}+\Delta A_{700\left(pH\;4.5\right)\;}\;$$

(4) $$\Delta A_{rel}=\frac{\Delta A_{520\left(pH1.0\right)}+\Delta A_{700\left(pH\;1.0\right)}+\Delta A_{520\;(pH\;4.5)}+\Delta A_{700(pH\;4.5)}}{A}$$

$\Delta {MAPC}_{rel}=\Delta A_{rel}$—as errors can only occur during absorption measuring as all other values were obtained from the literature;

(5) $$\Delta {MAPC}_{abs}\left(\frac{mg}{L}\right)=\Delta {MAPC}_{rel}\times MAPC$$

Anthocyanins were expressed as cyanidin-3-*O*-glucoside equivalent (µg/mL of AE).

### 2.4. A Bacterial Cellulose Pellicle-Film Production and Purification

The cultivation of KMC in tea medium resulted in the production of floating film. When the film reached a uniform surface and a thickness of 4 mm, it was removed and sent for cleaning from microbial cells and their metabolites. For cleaning, the BC film is placed in a 0.5% NaOH solution for 30 min at 90 °C and periodically stirred. Afterward, it is washed with distilled water until the pH of the washing water reaches 6–7.

### 2.5. Analysis of Anthocyanin Load and Release Performance

The gravimetric method was used to determine the release of total liquid from hydrogels impregnated with the elderberry extract. Squares of dry fabric genuine kid leather 7 × 7 cm were placed on Petri dishes with a diameter of 9.5 cm and weighed on scales with an accuracy of 0.001 g. Squares of hydrogels 3 × 3 cm (a thickness of 0.4 cm) saturated with distilled water or 20% of AE were placed on the leather samples and weighed again. The dishes were covered with lids and incubated for 3 h at room temperature, weighing the leather with and without hydrogel every hour and removing the lid. The weight of the hydrogel was calculated as the difference between the weight of the Petri dish with leather and hydrogel and the weight without hydrogel.

The coefficient of liquid loss through the film was determined by the Equation (6):

*K* = *A*_0_ − *Ax/A*_0_ × 100% (6)

where *A*_0_ is the weight of the hydrogel at the initial point, and *Ax* is the weight of the hydrogel at the time point *x*. There were three repetitions from two separate experiments.

To determine the load of AE into BC hydrogels, BC films were cut into squares of 3 × 3 cm (a thickness of 0.4 cm) and put into a Petri dish filled with 8 mL of elderberry extract to saturate with elderberry AE within three hours. The BC/AE samples were rinsed in distilled water, and extra water was removed using sterile textiles before further treatment. Absorption of C-3-*O*-glu was measured on wavelength spectra of AE before and after exposure of BC hydrogel to 20% AE. The concentration of C-3-*O*-glu was calculated via the Equation (7):

*MAPC* = (*A* × *MW* × 1000)/(*ε* × *l*) (7)

where the dilution factor was ignored due to its insignificance in these results.

The release AE rate was determined in three replicates by exposing uploaded samples to distilled water (pH 5.4) for three hours at room temperature. After that, the absorption of the resulting water was measured, and the anthocyanin concentration was calculated, as mentioned above.

### 2.6. Biological Activity Characterization of BC/AE

#### 2.6.1. Antimicrobial Activity of Hydrogels with Elderberry Extract

The antimicrobial activity of BC hydrogels saturated with AE was tested against gram-positive cultures *B. subtilis* and *S. aureus*, gram-negative *E. coli* and *P. aeruginosa*, and yeast *C. albicans* on LB agar medium in triplicate. Phosphate-citrate buffer (pH 5) was used to dilute the alcohol extract of elderberry fruits. The resulting solutions impregnated the BC films, and the latter were placed on the lawns. The saturation time of the hydrogels was one hour. BC hydrogel samples had the same dimensions of a 0.8 cm diameter and were sterilized by autoclaving at 0.5 atm, 112 °C, 30 min. A virgin BC hydrogel served as a control.

Undecylenic acid (UA, 98%) was supplied from Sigma-Aldrich (St Louis Mo, Missouri, USA). UA is a natural fatty acid obtained by pyrolysis of ricinoleic acid from castor oil. It has antimicrobial activity [45], and it was selected as the compound that did not discolor ANC pigments. Halochromic BC/ANC films (d = 0.8 cm) with AE were immersed in a 1% solution of UA in dimethyl sulfoxide (DMSO) (Sigma-Aldrich, St Louis, MO, USA).

#### 2.6.2. Cytotoxicity Assay

Cell viability was determined spectrophotometrically using the thiazolyl blue tetrazolium bromide (MTT) assay. The viable cell number was proportional to the production of water-insoluble formazan from MTT. The absorbance was measured spectrophotometrically at 570 nm after the dissolution of formazan crystals in DMSO [46]. Gifted from IMBG collection, human mesenchymal stem cells were previously isolated from the umbilical cord (UC-MSC) using an institutionally approved protocol. UC-MSCs were harvested at passage four and used at 5000 cells/well in DMEM-F-12 medium (Thermo Fisher Scientific, Waltham, MA, USA) supplemented with 10% FBS (fetal bovine serum) (Sigma-Aldrich, USA), and 1% penicillin/streptomycin (Thermo Fisher Scientific, Waltham, MA, USA). The culture was maintained in an incubator at 37 °C with 5% CO_2_. Human embryonic kidney cells HEK293 were obtained from the cell bank of the R. E. Kavetsky Institute of Experimental Pathology, Oncology and Radiobiology NAS of Ukraine. HEK293 cells were cultured as described above. Primary mouse fibroblast (PMF) cells were obtained from the soft tissues of 2-day-age mice of the ICR line. The isolated material was treated with ×10 penicillin/streptomycin (Gibco, Waltham, MA, USA) for 20 min, then with trypsin (0.20%) for 12 h, and finally, the solution was replaced with the DMEM-F-12 medium with FBS (10%). The PMFs were used at 5000 cells/well. After 24 h of incubation, the cells were treated with different doses of the AE in terms of anthocyanin content at an initial concentration of 240 µg/mL and with its dilutions by a factor of 10 to 2.4 × 10*^−^*^4^ µg/mL and incubated next 24 h. Afterward, the medium was replaced with a fresh one, and 20 µL of filter-sterilized MTT (5 mg/mL) in phosphate-buffered saline (PBS, pH 7.4) was added to each well and incubated at 37 °C for 3 h. The formed formazan crystals were solubilized with 100 µL of DMSO (Sigma-Aldrich, USA). The optical density of each well was measured using a microplate spectrophotometer (Tirertek Multiscan MCC340 (Flow Laboratories, North Ryde, Australia). Results were expressed as a % of untreated control. All experiments were repeated in triplicate.

#### 2.6.3. Scratch Assay (Activation of Mouse Fibroblast Migration and Proliferation)

Fibroblast cell proliferation and migration rates were determined using microscopic imaging and MTT test. An in vitro cell culture of PMF was used for the scratch test. Murine fibroblast cells were grown overnight in a 2% FBS medium and seeded onto the 12 well plates at a density of 90,000 cells/well. After 24 h of incubation at 37 °C, cells reached 80% of confluence, and the medium was discarded. Freshly prepared growth medium containing 2% FBS was added. After 24 h, the cells were scratched using a sterile pipette tip. Cells were washed with PBS, and a fresh 2% FBS medium was added. Different doses of elderberry alcohol extract, ranging from 2.4 µg/mL to 2.4 × 10*^−^*^3^ µg/mL, were added to the appropriate wells. The migration of cells towards the center of the gap after 24 h and 48 h of incubation after scratching was recorded by a camera in an inverted microscope Karl Zeiss, Primovert with ×4 magnification. Photographs of the scratched areas were analyzed using the ImageJ 1.54f/Fiji*^®^*. The results were calculated as the difference between the scratch area at 0 h, 24 h, and 48 h for each well, respectively. The experiment was performed in triplicate. After 48 h of fibroblast cell incubation, the MTT assay was used to record the difference in cell viability between variants. The *t*-test was used to estimate the differences between the scratch areas.

### 2.7. Physicochemical Characterization of BC/AE

#### 2.7.1. Fourier Transform InfraRed Spectroscopy

The absorption spectra of biofilm samples were obtained by FTIR to evaluate the macromolecular composition. The FTIR analyses were performed with a Bruker IFS-113v Fourier Transform Infrared spectrometer (Bruker Corporation, Billerica, MA, USA). Measurements were performed at room temperature in the range of 500–4000 cm^−1^ with a spectral resolution of 1.0 cm^−1^. The accuracy of determination of the line position was 1 cm^−1^. The BC/AE specimens of 3 × 3 cm (thickness 0.5 mm) in triplicate were dried at 50 °C overnight and used for analyses. Elderberry extract was smeared with a thin layer on the surface of the KBr plate, which was then placed inside the Fourier spectrometer and dried at a temperature of 35 °C for 24 h. An empirical crystallinity index was estimated using the absorption ratio at 1372 and 2900 cm^−1^ [47].

#### 2.7.2. Scanning Electron Microscopy (SEM)

For SEM, the BC/AE hydrogel samples (thickness 0.5 mm) in triplicate were dried at 50 °C for 6 h and contrasted with gold nanoparticles (10 nm). The samples were visualized using Quanta FEG-250 (Model No 1027641, FEI company, Brno, Czech Republic). The samples were analyzed at a magnification of 90,000 at a voltage of 20 kV. The width of the BC/AE membrane fibrils (n = 100) was measured by the ImageJ/Fiji*^®^* program. The *t*-test was used to estimate the differences between BC and BC/AE fibrils.

### 2.8. Smartphone-Based Sensor Systems Based on BC Hydrogels and pH-Indicators

BC hydrogels (d = 0.8 cm) were used as a sensitive element of a smartphone-based sensor system to control bacterial contamination. The BC hydrogel discs were incubated in the mixture (1:1 *v*/*v*) of the AE and 100 mM phosphate-citrate buffer solution with different pH (pH 4, 5, 6, 7) for 1 h. To evaluate the ability of the BC hydrogels with immobilized anthocyanins to change color in response to microbial contamination, the BC discs were placed on Petri dishes with bacterial lawns of *B. subtilis* and *P. aeruginosa*. The polymer film’s color change was recorded using the Samsung A52 smartphone camera. The smartphone was fixed at 10 cm from the samples under investigation using a 26 cm diameter 16 W ring LED lamp tripod. Digital images of the polymer films were captured every 10 min for 3 h. The intensity of the BC hydrogel disks’ staining and the appearance of a different color were detected using the mobile application Spotxel Reader 2.5.1 (SICASYS Software GmbH, Germersheim, Germany). The experiments were repeated in triplicate.

### 2.9. Animals

Pregnant ICR mice (body weight 19–23 g) were kept in special animal facilities at the Institute of Molecular Biology and Genetics. Animals were housed in quiet, temperature-controlled rooms (22–23 °C) with a 12 h light: 12 h dark cycle (lights on between 08:00 and 20:00 h). The animals were provided with purified water and dry food pellets ad libitum. The experimental procedures were carried out according to the standard ethical guidelines (European Community Guidelines on the Care and Use of Laboratory Animals 86/609/EEC) and approved by the Institutes’ Ethics Committees.

### 2.10. Statistical Analyses

The presented data were expressed as mean values ± SD (standard deviation), where each value was the average of three replications. The *t*-test was used to estimate the differences between groups. *p* values lower than 0.05 were considered to be significantly different. Statistical significance is indicated by asterisks (* *p* < 0.05, ** *p* < 0.01, *** *p* < 0.001, **** *p* < 0.0001).

## 3. Results

### 3.1. Black Elder Extracts, Appropriate Halochromic BC Hydrogels, and the Anthocyanin UV-VIS Spectra in the Range of pH of 4 to 12

**Figure 1:**
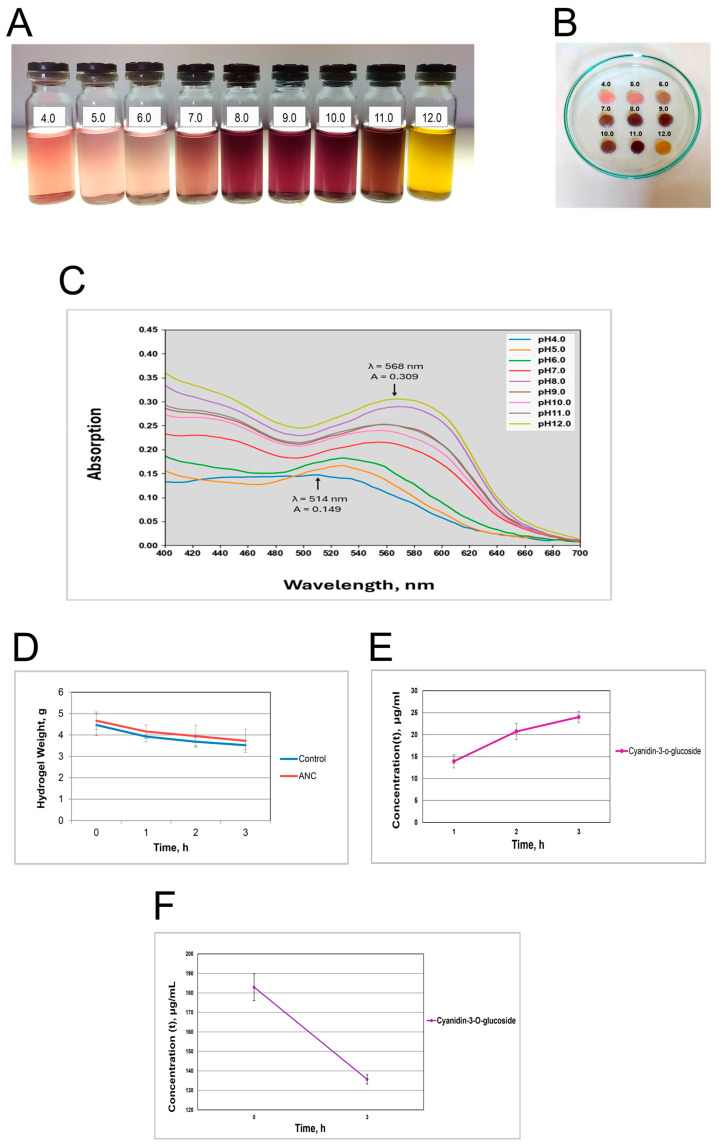
A pH-dependent color line of the elderberry fruit extract (pH 4–12) (**A**), bacterial cellulose (BC) hydrogels filled in the anthocyanin extract (AE) at pH from 4.0 to 12.0 (**B**), the anthocyanin UV-VIS spectra at pH 4–12 in the range λ 400–700 nm (**C**), dynamics of the cumulative liquid release through the BC/AE and BC (control) films into a model leather (**D**); the absorbance of anthocyanin extract by a hydrogel film (**E**), and the anthocyanin extract release from the hydrogel into a distilled water (**F**).

Phosphate-citrate pH buffers ranging from 4.0 to 12.0 were used to generate the pH line of AE solutions. Figure 1A shows that total anthocyanins cause a red color at pH 4, probably due to cyanidin 3-*O*-glucoside, which is predominant (87.80% of the anthocyanins) in elderberry fruits [48]. Then, at pH 5, 6, and 7, it changes into purple and turns dark blue at pH 8, 9, and 10, followed by khaki at pH 11. At pH 12, the color sharply changes to yellow. The same AE was used to saturate purified BC. Hydrogel test BC/AE samples had the same spectrum of colors as appropriate buffer solutions (Figure 1B). The anthocyanin UV-VIS spectra at pH 4–12 in the λ range 400–700 nm show that pH influences ANC absorption and intensity (Figure 1C).

At pH 4, anthocyanins of the red color absorb at 520 nm with an intensity higher than at pH 5–6 (Figure 1C). The increased pH to 5–6 and appropriate change in color led to a slight peak shift ranging from 520 to 530 nm. At pH 7 to 10, the absorbance peak shifted dramatically to 565 nm with a proper change in the extract’s color to violet shades. Due to structural modifications, the highest intensities and shifts were observed at pH 11–12, with a sharp color variation from violet to khaki and yellow.

As calculated using spectral data, the C-3-*O*-glu concentration in the used AE sample equals 427.29 ±8.015 (mg/L). C-3-*O*-glu concentration in the raw elderberry juice was estimated to be 1.5 ± 0.03 g/L. The anthocyanin content calculation in the AE sample is represented in Appendix A (Table A1).

### 3.2. Anthocyanins Upload and Release Performances

The liquid released from hydrogels impregnated with the elderberry extract into kid’s leather was observed for three hours (when the BC film/dressing is usually wet on a wound). The cumulative release ratio of anthocyanins from cellulose hydrogel was dependent on time: 10.80% (1 h), 15.37% (2 h), and 20.12% (3 h). The cumulative release ratio of water from hydrogels was 12.11% (1 h), 17.49% (2 h), and 21.18% (3 h) (Figure 1D; Table A2). There was no significant difference between releasing water and AE from BC/AE films. Based on the difference in concentrations of C-3-*O*-glu in the elderberry extracts before and after AE upload, the BC hydrogel has uploaded an average of 48 ± 2.43 mg/L of anthocyanin within 3 h (Figure 1E, Table A3). Results show that the amount of anthocyanins released from the BC/AE film after 3 h in distilled water (pH 5.4) is about 25 µg/mL (Figure 1F; Table A4).

### 3.3. Smartphone-Based Sensor System

**Figure 2:**
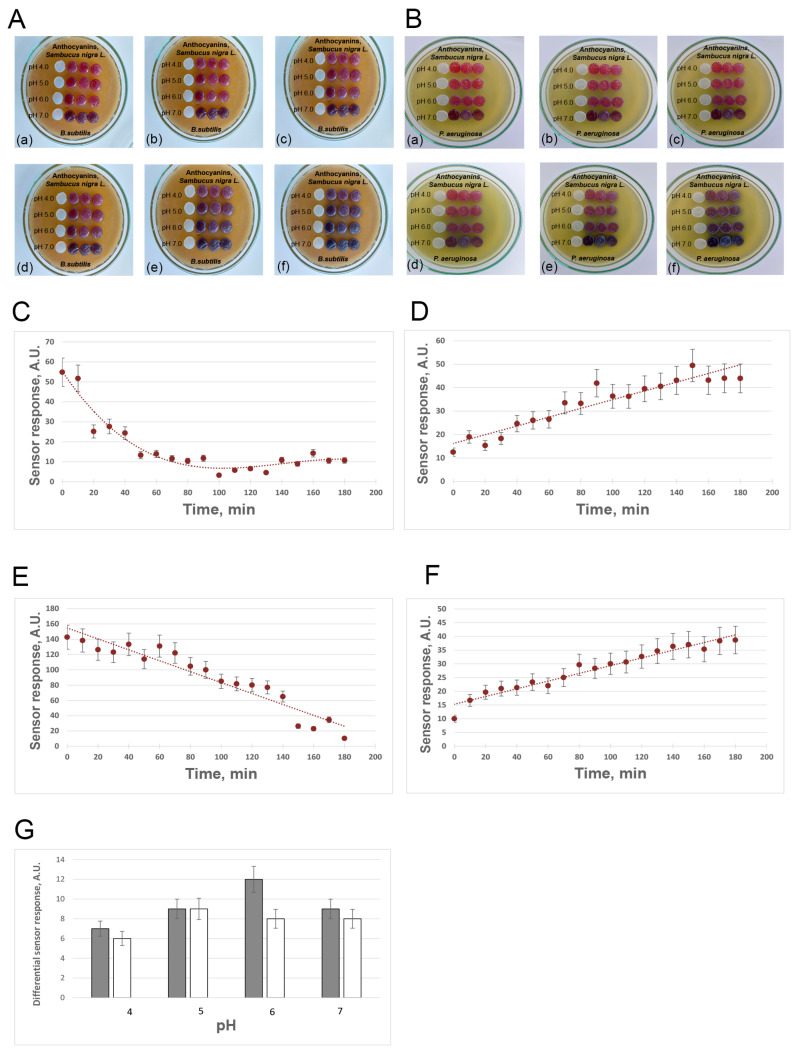
Smartphone-based sensor system based on bacterial cellulose (BC) hydrogel membranes and natural anthocyanins of black elderberry (*Sambucus nigra* L.) design. (**A**,**B**) changes in the color of the BC hydrogel disks with natural anthocyanins immobilized in their structure at the time of incubation on the *Bacillus subtilis* (**A**) and *Pseudomonas aeruginosa* (**B**) lawns and on pH of the 100 mM phosphate-citrate buffer used in immobilization procedure. Incubation time: 0 min (**a**), 20 min (**b**), 40 min (**c**), 60 min (**d**), 120 min (**e**), 180 min (**f**). (**C**,**D**) dependence of the value of the sensor responses (intensities of red (**a**) and navy blue (**b**) staining) of the smartphone sensor based on BC hydrogel membranes with the immobilized natural anthocyanins on the time of incubation on the lawn of *B. subtilis* (**C**,**D**) and *P. aeruginosa* (**E**,**F**). The 100 mM phosphate-citrate buffer solution pH 5.0 was used in the immobilization procedure. (**G**)—the dependence of the value of differential sensor responses (dark violet color intensity) of the BC hydrogel-based sensor membranes with natural anthocyanins on pH of the 100 mM phosphate-citrate buffer used in the immobilization procedure. The sensor membranes were incubated on lawns of *B. subtilis* (grey bars) and *P. aeruginosa* (white bars) for 30 min (**G**), the differential sensor response was calculated as the difference in the intensity of blue staining of the sensor BC hydrogel-based membranes before the contact with bacteria and after 30 min incubation on the bacterial lawns.

The BC hydrogels with natural anthocyanins immobilized in their structure at pH 4.0 and 5.0 in 100 mM phosphate-citrate buffer were able to change their color from pink-red to dark violet from the first minutes of incubation on the lawn with the *B. subtilis* (Figure 2(Ab)) or *P. aeruginosa* lawns (Figure 2(Bb)). These changes can be assessed visually (Figure 2A,B) and quantitatively using the Spotxel Reader 2.5.1 mobile application (Figure 2C–F). Pronounced color changes of the developed BC hydrogel-based sensor membranes from pink-red to dark violet were observed at a pH 6.0 of 100 mM phosphate-citrate buffer and less pronounced color changes—at pH 7.0. In this case, it was possible to register changes in the blue color intensity using a smartphone camera and the Spotxel Reader 2.5.1 application, but visually, these changes were much less pronounced as compared to the BC hydrogel-based sensor membranes with anthocyanins immobilized at pH 5.0 and 6.0. As seen from Figure 2G, the optimal pH values of 100 mM phosphate-citrate buffer are pH 5.0 and 6.0 since they result in the highest values of the further sensor responses.

The smartphone-based sensor’s limit of detection (3 σ) showed that all sensor signals with a value exceeding 3.45 A.U. were associated with the activities of bacterial metabolites.

### 3.4. The Low Antimicrobial Activity of Hydrogels Impregnated with Elderberry Extract Is Shown

**Figure 3:**
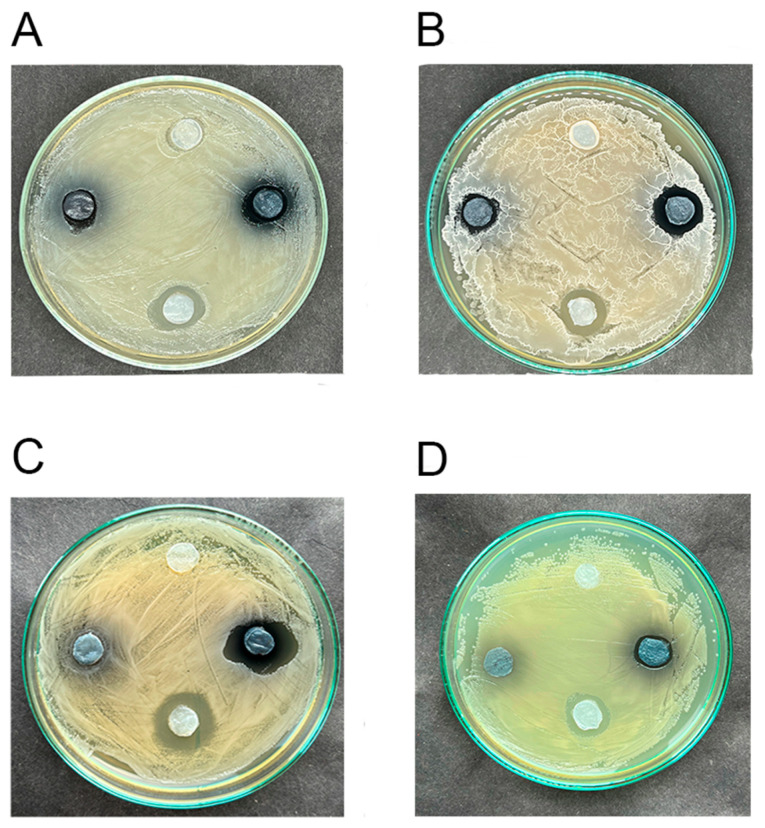
Antimicrobial activity of hydrogels with the elderberry anthocyanin extract (AE) and undecylenic acid (UA). (**A**) *Staphylococcus aureus*; (**B**) *Bacillus subtilis*; (**C**) *Candida albicans*; (**D**) *Escherichia coli* lawns, where the cellulose-based hydrogel discs impregnated with AE (on the left) and +UA added (on the right) placed and incubated 16 h. As controls, pure hydrogel (on the top) and hydrogel saturated with UA without AE (on the bottom).

BC-hydrogels impregnated with elderberry AE in phosphate-citrate buffer (pH 5) showed weak antimicrobial activity against gram-positive bacteria and yeast *C. albicans* (Figure 3A–C). Antibacterial activity against gram-negative bacteria *E. coli* and *P. aeruginosa* was not detected, probably because of its low rate (Figure 3D, Figure S1). Notably, black elder extract inhibited the synthesis of a siderophore pyoverdine by pseudomonad cells (Figure S1), as have been shown previously [49,50].

### 3.5. Hydrogels Loaded with the Elderberry Extract and Undecylenic Acid Exhibit More Potent Antimicrobial Activity than the Extract Alone

The photograph (Figure 3) shows that the BC discs with elderberry extract and UA produce more pronounced zones of inhibition of microbial growth after 16 h of incubation than BC/AE without UA. This was evident in suppressing representatives of *Candida*, *Staphylococcus*, and *Bacillus* genera (Figure 3A–C). *E. coli* was not suppressed by AE, but UA inhibited its growth, as previously reported for this bacterium [45]. The color of the discs changed from pink-red to gray-blue within an incubation time, indicating the alkalization of the culture media.

### 3.6. The Absorption Spectra of Biofilm Samples Proved the Presence of Flavonoid Rings

**Figure 4:**
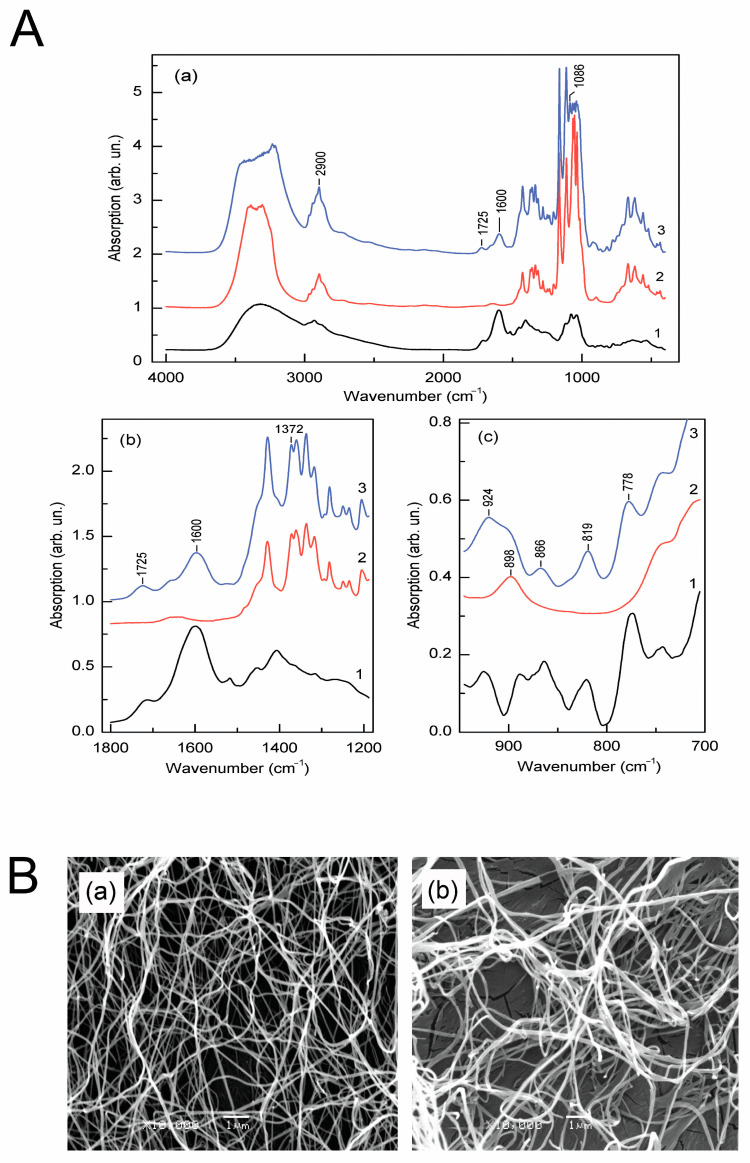
Characterization of bacterial cellulose (BC) films loaded with anthocyanin extract (AE) with Fourier-transform infrared spectroscopy (FTIR) spectral analysis (**A**) and scanning microscopy analysis (**B**). (**Aa**), the FTIR spectra of BC/AE. (**A**(**b**,**c**)), spectra fragments demonstrating AE’s most pronounced influence on BC. 1, 2, 3—spectra for AE, BC, BC/AE, accordingly. (**Ba**), an SEM image of the purified BC’s nanostructure; (**Bb**), an SEM micrograph of the BC loaded with AE. Scale bar: 1 μm. The mean width for BC microfibrils is 113 ± 28 nm; the mean width for BC/AE microfibrils is 172 ± 58 nm. Mean values are significantly different (*p* < 0.05).

Anthocyanins are a class of water-soluble flavonoids with an esterified carbohydrate. The flavonoid skeleton has three rings and the charged oxygen atom on the C ring. The following functional groups are typical for anthocyanins: C-H (aliphatic, aromatic), O-H, and C=C (aromatic). In Figure 4(Aa), the absorption spectra of elderberry extract, cellulose, and BC-based film impregnated with elderberry extract are shown, and in Figure 4(Ab,Ac), fragments of the spectra that demonstrate the most pronounced influence of elderberry extract on BC and, accordingly, on the structural changes of cellulose are represented. In particular, the FTIR data of BC/AE show a broader than in purified cellulose band of absorbance between 3500 to 3000 cm^−1^, demonstrating O-H stretching vibrations (Figure 4(Aa)). In the spectrum region of 1750–1525 cm^−1^, the appearance of two new broad absorption bands with maxima at 1725 and 1600 cm^−1^, characteristic of elderberries, is observed (Figure 4(Ab)). The broad absorption band at 1603 cm^−1^ is attributed to the aromatic C=C, and 1725 cm^−1^ is to the C=O stretching characteristic of polyphenols [51].

In the region of 1100–750 cm^−1^, which is characteristic of C-O (C-O-C) stretching and C-H bending vibrations of ANCs, the appearance of additional absorption peaks at 1086, 920, 866, 819, and 778 cm^−1^ is observed (Figure 4(Ac)). The vibrational mode at 1086 cm^−1^ can be identified as the stretching asymmetric mode C-O-C group in ethers. The 819 cm^−1^ band is associated with C-O-C stretching symmetric vibrations. An additional absorption band at 924 cm^−1^ is attributed to C=C bending, alkenes. Two vibrational modes at 866 and 778 cm^−1^ are associated with C-H bending of aromatic hydrocarbons in 6-membered aromatic rings. Notably, characteristic for cellulose monomer—glucose—a band with a maximum at 898 cm^−1^ (Figure 4(Aa2)) broadens significantly, and its symmetry changes in a hybrid cellulose polymer (Figure 4(Aa3)). Component decomposition of the band showed that it consists of two components with maxima at 898 cm^−1^, and an additional absorption component appears with a maximum at 924 cm^−1^ that coincides with the vibrational mode in elderberry extract (Figure 4(Aa1)).

Despite the changes in the IR spectrum of the BC-based film impregnated with elderberry extract, the main signs of its crystallinity are preserved. Nevertheless, the BC/AE crystallinity estimated by the intensities of the absorption components I372/2900 decreased by 13.5% in the BC/AE film.

Based on the obtained results, the following scheme of possible physicochemical interactions between anthocyanins and BC could be suggested (Figure S2). Thus, polar phenolic and hydroxyl groups in the anthocyanin structure create numerous possibilities for forming hydrogen bonds with BC macromolecules.

### 3.7. Scanning Electron Micrographs Show Thickened Cellulose Nanofibrils in Films Filled with Elderberry Extract

The SEM micrographs of pure BC film showed an extensively entangled fibril network with irregular fibril void arrangement Figure 4(Ba). Nevertheless, the BC/AE fibrils’ structure in Figure 4(Bb) shows altered morphology. In particular, the width of the microfibrils increased significantly from 113 ± 28 nm (pure cellulose) to 172 ± 58 nm. The highly bundled BC fibril network was less dense in the BC/AE film, and pores were more prominent than in BC.

### 3.8. Cytotoxicity Assay Shows Working Anthocyanin Concentrations

**Figure 5:**
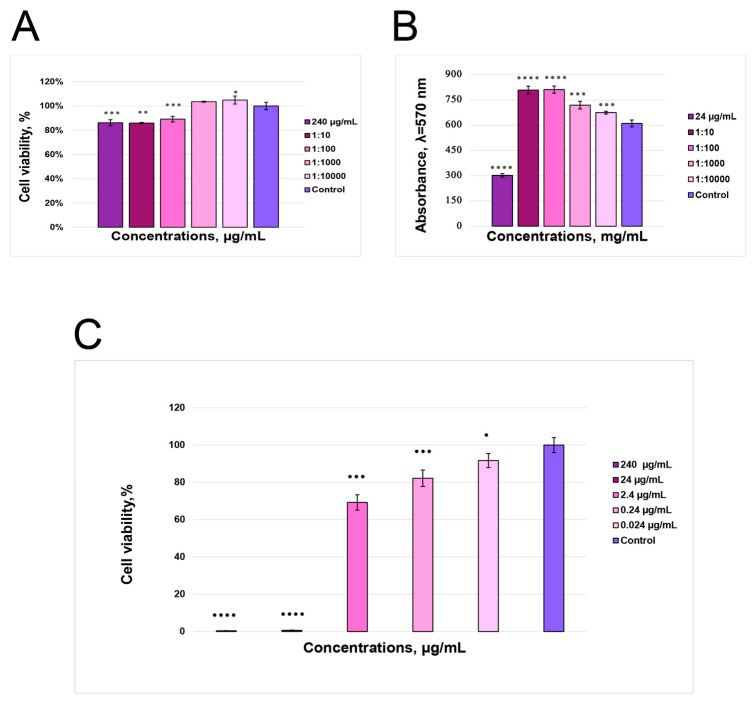
Effects of elderberry anthocyanin extract on the viability of mesenchymal stem cells from the umbilical cord (**A**), primary mouse fibroblast cells primary mouse fibroblast cells primary mouse fibroblast cells (**B**), and human embryonic kidney cells (**C**). The MTT assay determined the cytotoxicity data, as mentioned in Materials and Methods. The error bars represent the standard deviation of the mean (SD). Statistically significant changes in treatment groups compared with control are indicated by asterisks (* *p* < 0.05; ** *p* < 0.01; *** *p* < 0.001; **** *p* < 0.0001).

The MTT test for UC-MSC showed that the concentrations of ANC in AE (2.4; 24.0; 240.0 µg/mL) inhibited MSC proliferation by 11–15%. However, UC-MSC did not exhibit cytotoxicity (Figure 5A). The diluted elderberry extract with anthocyanin content (0.24 µg/mL) did not affect MSC growth. Contrary to MSC, the HEK293 cell line exhibited 24.0 and 240.0 µg/mL doses to be toxic (Figure 5C). In experiments with PMF, the MTT test revealed that a dose of ANC 24.0 µg/mL reduced cell viability to 37% compared to control cells; however, lower ANC concentrations stimulated cell growth (Figure 5B).

### 3.9. In Vitro Fibroblast Migration Assay

**Figure 6:**
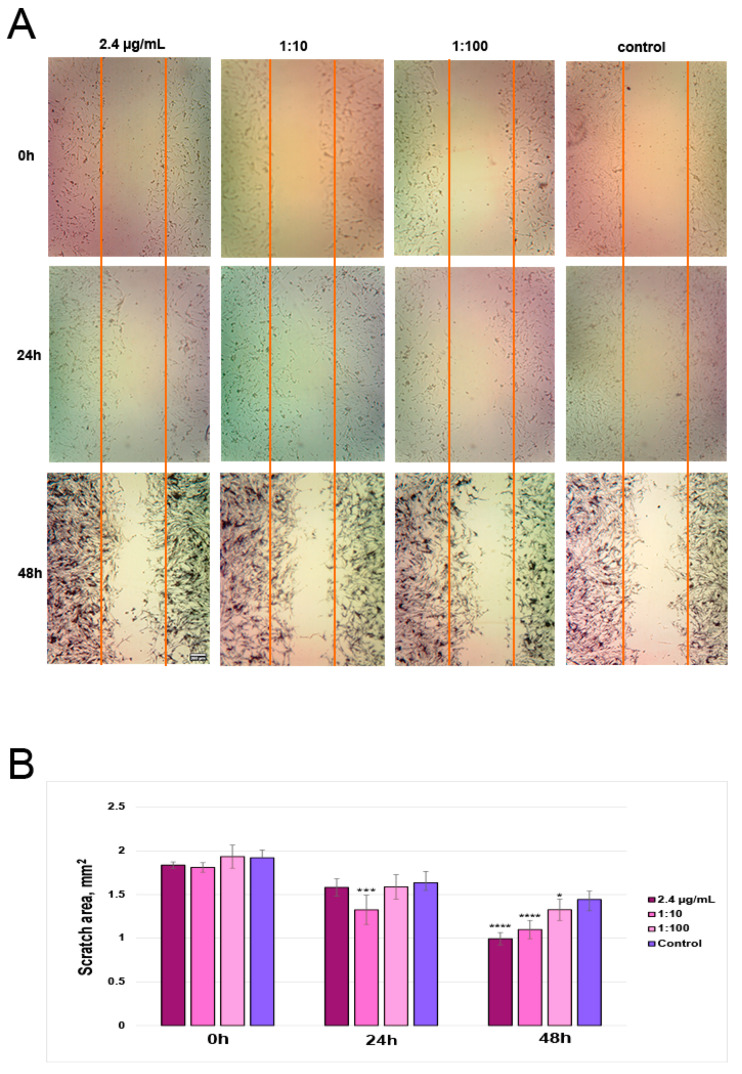
In vitro fibroblast migration assay results. (**A**) microscopic images showing a scratched area at the beginning of the experiment (0 h) and after 24 and 48 h of treatment; (**B**) scratched areas calculated between scratch edges before and after the treatment in different periods. The scratch and MTT assay determined were performed, as mentioned in Materials and Methods. The error bars represent the standard deviation of the mean (SD). Statistically significant changes in scratched areas compared with control are indicated by asterisks (* *p* < 0.05; *** *p* < 0.001; **** *p* < 0.0001).

A photo of mouse primary fibroblast migration rates under the effect of AE shows that after 24 h, the 0.24 µg/mL ANC dose reduced a square between the edges of the scratch faster compared to the control cell line, as seen in Figure 6A. This means that PMFs were activated with AE and filled in the scratch area faster. After 48 h, ANC-equivalent doses of 2.4–0.024 µg/mL showed the effect of fibroblast activation (Figure 6B).

## 4. Discussion

Here, a prototype of the dual-use smart hybrid film has been designed as a potential agent for wound/burn healing and as a halochromic sensor for microbial contamination monitoring. The hybrid film consists of hydrated cellulose polymer as a matrix produced by the bacteria and the extract of *Sambucus nigra* L. berries as a pH sensor. Bacterial cellulose is a competitive polymer among natural ones for wound/burn dressing design as it is readily available, nontoxic, biocompatible, occlusive, prevents scar formation, and is easily disposable. Recently, BC was saturated with mesoporous silica nanoparticles, which served as a shell for pH-sensitive synthetic dye, to design a halochromic wound dressing [52]. In this study, BC was an excellent matrix for a natural pH sensor dye (anthocyanins) while also acting as a potential wound-healing agent. For the first time, we combined eco-friendly products to fabricate a wound/burn dressing prototype, elderberry fruit extract and bacterial cellulose, in the hybrid hydrogel, which has dual uses for would/burns treatments and online microbial contamination monitoring.

The known disadvantage of halochromic sensors is the rapid dye leaching into the wound. Several solutions to this problem have been found, e.g., Tamayol et al. [53] used hydrogel fibers containing mesoporous polyester beads with encapsulated dye to protect the dye from fast leaching into the wound. Liu et al. [54] reinforced alginate hydrogel with a covalently cross-linked polyacrylamide network and incorporated the pH-sensitive dye phenol red. In this study, we resolved this problem using cellulose matrices/dressings with a natural dye, anthocyanins, which both contain active functional groups. These allow them to interact and establish unstable connections, preventing rapid ANC-dye leakage. We showed experimentally that within a period when the cellulose film was wet on top of the injured skin (approximately three hours), the release of anthocyanins was 20% of the uploaded content in the BC film. Notably, this average AE output is not toxic for the in vitro cell lines and is pretty enough to activate skin fibroblasts.

Incorporating anthocyanins into various matrices is a widespread practice; however, it often causes multiple changes in the properties of the films, including their stability, antioxidant or antibacterial traits, etc., sometimes worsening their mechanical characteristics [55]. In principle, the morphology of a cellulose polysaccharide can be altered after specific treatments, e.g., exposure to unique stressors [56]. Therefore, additional ingredients are required to stabilize the pH dye-loaded matrix. Our results show that the properties of bacterial cellulose polymer were altered after impregnation with elderberry extract, e.g., the diameter of microfibrils increased. Understanding the mechanisms of interactions of BC polymer with elderberry anthocyanins was essential to predicting the stability of the hybrid polymer. For this, FTIR spectroscopy analysis provided valuable information on BC-ANC interactions. After examining the absorption spectra of BC and hybrid polymer, it was concluded that these interactions had a minimal impact on their molecular structure and biological activities. In this study, the FTIR results confirmed the presence of flavonoid groups in BC/AE samples; simultaneously, modification of some signals (i.e., the shift of peak of phenols) has been detected. Earlier, it was known that noncovalent interactions between polyphenols and plant polysaccharides were mainly due to weak associations formed due to a combination of hydrogen bonds and hydrophobic interactions [57]. It was also showed that cationic ANCs had the adsorption capacity and binding affinity to plant cellulose, attaching directly to the electronegative hydroxyl groups of cellulose fibrils due to noncovalent coulombic interactions, as well as hydrogen bonding between the anthocyanin and cellulose [58,59]. This study used a natural dye, elderberry fruit extract, which contains anthocyanin molecules with active functional groups (see Figure S2). Due to the latter, ANCs can interact with the cellulose matrices/dressings to prevent fast leakage of releasing ANCs. We revealed that ANCs released slower than water from BC hydrogel impregnated with therapeutic doses of ANC, probably due to unstable interaction with the polysaccharide polymer. Therefore, polar phenolic and hydroxyl groups in the anthocyanin structure create numerous possibilities for forming hydrogen bonds with BC polymer. Such weak linkages provide a controlled anthocyanins’ release and preserve their biological activities. Our results also show that thickened bacterial cellulose fibrils could be attributed to the BC functional OH-groups’ extensive interactions with ANC groups.

In a previous study, anthocyanins loaded on cellulose acetate ultrafine fibers reduced cellulose crystallinity [55]; also, the crystallinity index of the cellulose film decreased with the addition of anthocyanins and oregano essential oil [60]. In this study, the crystallinity index of BC was slightly reduced after the impregnation of elderberry extract. The increased cellulose amorphousness was translated into forming larger pores and a less dense fibril network in the BC/AE film, which could be advantageous under uploading performances.

The prototype of hydrogel BC/AE dressing is a promising candidate for in situ wound contaminants diagnostics and wound/burn therapy as it exhibited efficacy in the in vitro assays and meets the WHO/REASSURED criteria of being accessible, sensitive, specific, user-friendly, rapid, equipment-free, affordable, real-time monitored, and easy to sample [61]. Selective elements of sensor devices for designed BC/AE films changed color from pink-red to dark violet at pH 4–6 upon contact with various bacteria on solid surfaces within minutes. The color changes were successfully recorded visually. With image analysis software installed on a smartphone, it is easy to detect invisible changes in color/pH/bacterial contamination. In addition, pH-dependent color dressings can be disposed of quickly and safely as both cellulose and elderberry components are biodegradable. These cellulose-polyphenol(anthocyanin) system features provide an advantage over chemically synthesized matrices [30]. The healing potential of anthocyanin extracts is mainly due to their antioxidant and anti-inflammatory properties. The antimicrobial activity was detected, but to enhance it, the natural user-friendly compound, undecylenic acid, was added to BC/AE films; we observed that the effect was more likely to be additive. Both BC hydrogels loaded with neat undecylenic acid (BC/UA) and BC hydrogels loaded with the elderberry anthocyanin extract (BC/AE) possess lower antimicrobial activity than BC/AE/UA film.

After a series of preclinical experiments, the prototype dressing can be successfully used in translation medicine. Our results showed that there could be therapeutic nontoxic doses for wound/burn healing, which promote fibroblast movement. Toxic doses are probably available for skin cancer treatment, as anthocyanins are known anticancer agents [62]. The scratch in vitro healing test is the earlier assay, indicating promising results for BC/AE/UA films. The following steps will include a study of the expression of tissue regeneration markers and in vivo tests using animals. The dual-use hybrid BC/AE/UA dressing could be competitive in hospitals, burn centers, and cosmetic clinics to diagnose microbial contamination during primary skin injury treatment. At the same time, further research is needed to design multiplexed theranostic smart dressings to diagnose and heal chronic wounds [63].

## 5. Conclusions

A dual-use smart hydrogel dressing prototype designed for in situ monitoring bacterial contamination in wounds possesses a stable presence of anthocyanins of *Sambucus nigra* L. in the BC hydrogel composition, probably due to the interaction of its functional groups with OH-groups of cellulose. The presence of anthocyanins in the BC polymer slightly altered the morphology and network of its fibrils. Anthocyanins sense a change in the pH of the microenvironment, and the color changes of the ANC sensor elements can be successfully recorded visually or with the help of commercially available digital image analysis software installed in a smartphone. The elderberry extract is not toxic for mouse fibroblasts at concentrations released from BC/AE dressing, and small AE doses stimulate their migration. The activation of mouse fibroblasts at therapeutic concentrations may predict their healing capability. Combining natural dye-pH-sensor with healing potential, the BC/AE hybrid polymer could provide an attractive initial material for simultaneous wound healing and online monitoring of bacterial metabolites.

## Data Availability

Data supporting reported results are present in the manuscript.

## Supplementary Materials

The following supporting information can be downloaded at: https://www.mdpi.com/article/10.3390/polym16162327/s1, Figure S1. Elderberry fruit anthocyanins (ANC) inhibit the pyocyanin pigment produced by *Pseudomonas aeruginosa*. A, Bacterial cellulose hydrogel discs impregnated with ANCs, on the bacterial lawn (0 h); B, after 16 h, halochromic discs change a color; no pigment is seen on a bacterial lawn around halochromic discs; control cellulose discs are surrounded with the pyocyanin. Figure S2. Possible physicochemical interactions between functional groups of anthocyanins and bacterial cellulose polymer.

## Appendix A

### Anthocyanin Content in the Sample Calculation

The absorbance of the diluted sample following the formula mentioned in the Materials and Methods section equals 0.6406. Having received the absorption, it was possible to obtain MAPC (monomeric anthocyanin pigment concentration—for elderberry extracts, C-3-*O*-glu is mentioned to be dominant), which was 427.29 mg/L, where A = 0.6406—absorption of the sample, MW = 449.2—C-3-*O*-glu molar weight, DF = 4—dilution factor, ε = 26,900 L/mol·cm—C-3-*O*-glu molar absorptivity, l = 0.1 cm—spectrophotometer path length. Errors calculations are represented in Table A1.

**Table A1:** Statistical data on Δ (A abs)—absolute instrumental error; Δ (A rel) = Δ (A abs)/A—absorption relative error; Δ (MAPC rel) = Δ (A rel)—monomeric anthocyanin pigment concentration relative error; Δ (MAPC abs) (mg/L) = Δ (MAPC rel)/MAPC = 8.015—monomeric anthocyanin pigment concentration absolute error.

Δ (A abs)	0.012
Δ (A rel)	0.018
Δ (MAPC rel)	0.018
Δ (MAPC abs) (mg/L)	8.015

**Table A2:** The cumulative release rate of the elderberry extract from cellulose hydrogel.

Time, h	Reference Hydrogel		Hydrogel with the Elderberry Extract	
	Weight, g	Coefficient of Water Release, %	Weight, g	Coefficient of the Extract Release, %
0	4.467 ± 0.502	0	4.666 ± 0.408	0
1	3.926 ± 0.248	12.110	4.162 ± 0.315	10.802
2	3.686 ± 0.208	17.489	3.948 ± 0.513	15.377
3	3.521 ± 0.222	21.176	3.727 ± 0.547	20.116

**Table A3:** The load rate of anthocyanin extract into cellulose hydrogel.

Time, h	Absorption, 517 nm	C-3-*O*-Glu Concentration, µg/mL
0	1.1 ± 0.04	182.9 ± 6.9
3	0.812 ± 0.02	135.7 ± 2.4

**Table A4:** The release rate of anthocyanin extract from uploaded cellulose hydrogel.

Time, h	Absorption, 517 nm	C-3-*O*-Glu Concentration, µg/mL
1	0.082 ± 0.01	13.9 ± 1.5
2	0.124 ± 0.01	20.7 ± 1.8
3	0.144 ± 0.01	24.0 ± 1.3
