# Supplementary material for: Halochromic Bacterial Cellulose/Anthocyanins Hybrid Polymer Film with Wound-Healing Potential
Source: Polymers (Basel). 2024 Aug 16;16(16):2327. doi: 10.3390/polym16162327 (PMC11359050; doi:10.3390/polym16162327)
Supplement: Supplementary file 1 [file polymers-16-02327-s001.zip › Suppolementary.pdf]

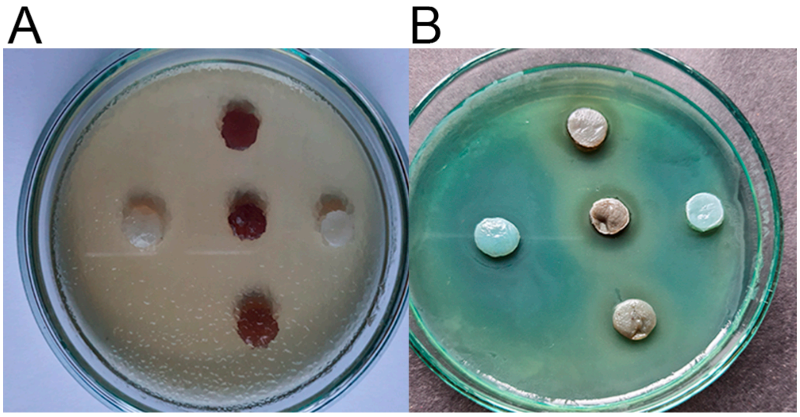

Figure S1. Elderberry fruit anthocyanins (ANC) inhibit the pyocyanin pigment produced by *Pseudomonas aeruginosa*. A, Bacterial cellulose hydrogel discs impregnated with ANCs, on the bacterial lawn (0 h); B, after 16 h, halochromic discs change a color; no pigment is seen on a bacterial lawn around halochromic discs; control cellulose discs are surrounded with the pyocyanin.

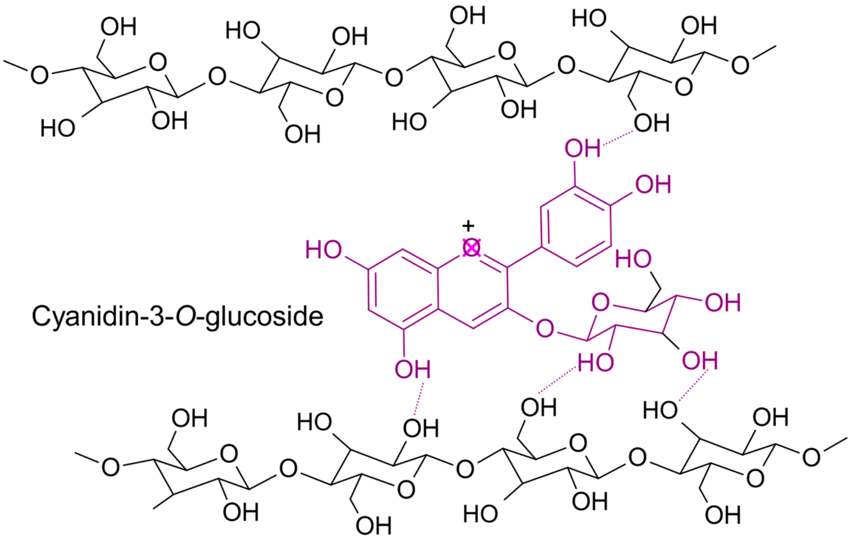

Figure S2. Possible physicochemical interactions between functional groups of anthocyanins and bacterial cellulose polymer.
